# Volatiles and Water- and Fat-Soluble Precursors of Saanen Goat and Cross Suffolk Lamb Flavour

**DOI:** 10.3390/molecules18022150

**Published:** 2013-02-07

**Authors:** Marta Madruga, Ingrid Dantas, Angela Queiroz, Luciana Brasil, Yuri Ishihara

**Affiliations:** Post-Graduate Program in Food Science and Technology, Technology Centre, Federal University of Paraiba (UFPB), João Pessoa-PB, CEP 58051-900, Brazil; E-Mails: ingridcdantas@hotmail.com (I.D.); angelalimas@gmail.com (A.Q.); luciana-vet@uol.com.br (L.B.); yuriufpb@yahoo.com.br (Y.I.)

**Keywords:** amino acids, fatty acids, goat, lamb, sugars, volatiles

## Abstract

This paper evaluates the concentrations of water- and fat-soluble precursors of meat flavour, with the aim of characterising the effect of species on the volatile profile of grilled goat and lamb meat. Compared to goat, lamb meat had higher levels of saturated fatty acids—SFA, monounsaturated fatty acids—MUFA and polyunsaturated fatty acids—PUFA and similar levels of sugars and free amino acids, except for lysine and glycine, which were higher in goat. Major differences were detected in lipid-derived volatiles; only pyrazine, thiazole, and some Strecker aldehydes were at different concentrations in these species. Volatile compounds derived from the oxidation of linoleic acid were at higher levels in meat from lamb due to the higher concentration of the latter, while compounds formed from α-linolenic acid were at higher levels in goat. It can be concluded that lamb meat has a stronger flavour profile compared to goat meat because it has the highest concentrations of lipid-derived volatile compounds, primarily straight saturated alkanals, pyrazines and thiazole.

## 1. Introduction

Sheep are important meat-producing animals worldwide, whereas goats are more important meat animals in the tropics [1,2,3]. Consequently, compared to sheep and cattle, knowledge of the aromatic quality of goat meat is limited due to the traditionally low economic significance of goat in developed countries [1]. However, goat meat has become popular and more acceptable in these countries due to its nutritional and sensorial parameters [4]. Goat has a low fat content (2.5 to 7.5 g/100 g) and a pronounced and distinguishable aroma [5,6].

Consumers tend to evaluate the quality of cooked meat on the bases of flavour, juiciness and tenderness; among these parameters, flavour and tenderness are the most important attributes that determine meat quality [7]. The majority of studies which compare the quality of goat meat and lamb meat only evaluate the organoleptic characteristics and the physical-chemical quality of these meats; none of these studies compare the volatile profile of these two types of meat [8,9,10,11].

The effect of species on the aromatic quality of goat and lamb meat is debatable because studies have reported controversial results related to flavour or odour intensity. For example, [12,13], reported that they could not find any significant differences in flavour between goat and lamb meat. However, [14,15] found that the meat flavour descriptors “goaty” and “muttony” were clearly distinguishable between goat and sheep, concluding that each meat had a specific species flavour. Furthermore, papers that reported flavour differences among species emphasised that the overall flavour intensity of sheep was stronger than goat, and concluded that goat meat is unique and is not interchangeable with meat from sheep. In general, these papers explain that differences in the flavour of goat and lamb meat may be a reflection of differences in the composition of fat depots [15,16,17].

Apart from studies of sensory and chemical quality involving the comparison of these two meats, no papers have been published which compare the volatile profile or the flavour precursors. In fact, several studies identified a number of variables influencing meat volatiles of lamb meat: for example, diet [18,19,20,21], breed [19], and castration [19], but only few papers studied goat meat [22,23]. Therefore, the aims of this study were to characterise the effects of the species (goat and lamb) on the volatile profile and the concentration of water- and fat-soluble precursors.

## 2. Results and Discussion

### 2.1. Fatty Acids

The fatty acid content of *Longissimus dorsi* samples from Saanen goat and Suffolk lamb is shown quantitatively (mg/100 g) in Table 1. Most fatty acids studied showed statistically different when comparing the two species. With the exception of linolenic (C18:3*n*3), docosapentaenoic (C22:5*n*3), and docosahexaenoic acids (C22:6*n*3), all of the fatty acids were at higher concentrations in lamb meat than in goat, with average concentrations of total fatty acid, SFA, and MUFA being 5-fold higher in Suffolk lamb meat. The PUFA concentrations were approximately 2-fold higher in lamb compared to goat. The results showed that fatty acid concentrations in Suffolk lamb meat were in accordance with published values [18,19].

**Table 1:** Fatty acid content of *Longissimus dorsi* from Saanen goat and cross Suffolk lamb.

	mg/100 g			
	Lamb	Goat	*P* ^1^	Standard error
C10:0	3.2	0.2	*	0.5
C12:0	4.0	1.1	*	0.5
C14:0	71.4	16.2	*	9.5
C15:0	14.9	4.2	*	2.1
C16:0	714.4	135.9	*	92.1
C17:0	53.1	9.4	*	8.1
C18:0	796.5	160.8	*	117.5
C19:0	3.1	2.3	NS	0.1
C20:0	3.0	1.5	*	0.3
C22:0	1.0	1.0	NS	0.1
C24:0	4.0	9.7	*	0.8
C14:1 *n*9c	2.7	0.0	NS	0.4
C16:1 *n*9t	14.7	4.7	*	1.9
C16:1 *n*9c	83.8	11.3	*	11.3
C17:1 *n*10c	28.2	4.6	*	3.7
C18:1 *n*6,8t	6.7	1.3	*	1.0
C18:1 *n*9t	7.5	1.1	*	1.0
C18:1 *n*10t	16.2	2.0	*	2.5
C18:1 *n*11t	23.3	17.2	NS	2.6
C18:1 *n*6,12c-t	8.5	0.9	*	1.3
C18:1 *n*13,14t	0.0	2.9	NS	0.4
C18:1 *n*9c	1380.0	209.4	*	172.2
C18:1 *n*11c	6.7	0.7	*	1.0
C20:1 *n*8,9c	0.5	0.0	NS	0.1
C20:1 *n*11c	3.0	0.5	*	0.4
C22:1 *n*13c	0.4	0.0	NS	0.1
C18:2 *n*9, 12 t-t	5.1	1.3	*	0.6
C18:2 9, 12 c-c	140.2	46.0	*	14.4
C20:2 *n*11,14 c-c	1.0	0.1	*	0.1
C20:3 *n*8,11,14c	3.9	2.4	*	0.2
C22:2 *n*13,16c	0.5	0.9	NS	0.1
CLA				
C18:3 *n*6	1.9	0.4	*	0.2
C18:3 *n*3	8.6	10.0	*	0.4
C20:4 *n*6	61.1	26.5	*	5.0
C22:4 *n*6	7.0	1.1	*	0.9
C22:5 *n*3	8.2	14.7	*	0.9
C22:6 *n*3	1.8	3.9	*	0.3
Total Fames	3601.4	736.6		452.3
UFA ^2^	1821.3	364.1		
MUFA ^3^	1582.0	256.7		
PUFA ^4^	239.3	107.4		
SFA ^5^	1668.6	342.3		
P:S	0.2	0.3		
*n-6:* n*-3*	3.8	1.0		

^1^ Probability that there is a difference between means; NS, no significant difference between means (*p* > 0.05); * significant at the 5% level; ^2^ Unsaturated fatty acids; ^3^ Monounsaturated fatty acids; ^4^ Polyunsaturated fatty acids; ^5^ Saturated fatty acids.

High proportions of Unsaturated Fatty Acids—UFA may be attributed to the lean carcasses produced from lamb and goat. Ruminant animals have been shown to preferentially deposit PUFA and MUFA in phospholipids rather than in neutral lipids. Enser *et al.* [24] reported higher concentrations of C20:5*n*3, C22:5*n*3, and C22:6*n*3 PUFA in lamb compared to beef and pork.

It is worthwhile to mention that in general, the total fatty acid amounts in our study are similar to those reported by other works comparing the quality of goat and lamb meat [16,17], as well as those studies that report the fatty acid profile of Suffolk lamb meat [18,19].

The major fatty acids were oleic (C18:1*n9c*), stearic (C18:0), palmitic (C16:0) and linoleic (C18:2n9,12c-c), which accounted for 84.3 and 74.7% of the total fatty acid in the *Longissimus dorsi* from goat and lambs, respectively. These results were in accordance with data reported in previous works comparing the fatty acid profile of goat lamb and meat quality [10,11,16].

As a result of their fatty acid profile, goat and lamb meat are ideal for health-conscious consumers [25]. The PUFA:SFA and the *Σn*-6:*Σn*-3 ratios, which are indicators of the nutritional quality of the lipids in a food, are in accordance with the values suggested by the U.K. Department of Health [26]. Furthermore, goat meat presents better fatty acid ratios; a PUFA:SFA value of 0.3 is close to the recommended value of 0.45, and the *Σn*-6:*Σn*-3 ratio of 1 is the ideal.

An interesting observation derived from these results is that the goat produces a leaner meat than lamb, which was expected, as goat meat has been reported previously as containing lower concentrations of fat and SFA [27].

### 2.2. Free Amino Acids and Sugars

The individual free amino acid contents of raw and cooked goat and lamb meat are shown in Table 2. No statistical difference was observed for most of the amino acids studied. The data for arginine are not present, as the analytical technique used is not suitable for arginine. Goat meat contained the highest concentrations of glutamine, alanine, glycine, glutamic acid, leucine, lysine, and total free amino acids compared with lamb, with a similar pattern for the remaining free amino acids. The most prominent free amino acids in both species were glutamine, alanine, glycine, glutamic acid, leucine, and lysine, which were present at concentrations greater than 10 mg/100 g.

Few data are available on the free amino acid composition of goat and lamb meat. When compared to our previous paper on goat meat [2], the concentrations of most of the free amino acids were in the reported ranges, except for cysteine, which was found in higher quantities in goat and lamb meat in the present study. Furthermore, Sheridan *et al.* [11] reported that Boer goat had significantly higher concentrations of eleven of the 18 essential amino acids that were measured than Merino lamb. Srinivasan and Moorjani [28] showed that goat meat contained more arginine, leucine and isoleucine than mutton.

No authors have previously compared the effect of cooking on the free amino acid composition of goat and lamb meat. The concentrations of all the amino acids were much reduced (40% to 60%) after grilling, although aspartic acid concentrations remained the same. The greatest reduction was observed in cysteine concentration for both species, a fact already for beef, by [29] for salmon, and by [30] in model systems.

**Table 2:** Mean concentrations (mg·100 g^−1^ wet weight) of free amino acids and sugars of *Longissimus dorsi* from Saanen goat and cross Suffolk lamb.

Water-soluble Precursors	Raw		*P*^1^	Cooked		*P* ^1^	Standard Error			Remaining %		
	Goat	Lamb		Goat	Lamb		Raw	Cooked		Goat	Lamb
*Amino acids*											
Cysteine	0.06	0.12	*	0	0		0.008	0.001		0	3
Methionine	0.6	0.7	NS	0.1	0.4	NS	0.067	0.042		19	51
Leucine	12.8	11.7	NS	5.1	4.8	NS	0.28	0.22		40	41
Isoleucine	5.6	5.4	NS	2.3	2.3	NS	0.12	0.11		42	42
Serine	4.5	4.3	NS	1.9	1.8	NS	0.09	0.09		42	42
Threonine	9.7	9.8	NS	2.9	3.8	NS	0.69	0.25		30	39
Valine	9.0	8.6	*	4.1	3.4	NS	0.40	0.18		46	40
Phenylalanine	5.9	5.5	NS	2.4	2.2	NS	0.07	0.11		41	40
Aspartic acid	5.0	6.2	*	4.9	5.8	*	0.54	0.50		98	92
Glutamic acid	14.2	10.4	*	5.3	5.5	NS	1.35	0.25		37	53
Glycine	31.5	20.3	*	19.7	10.5	*	3.26	1.14		63	52
Alanine	47.8	41.9	*	30.3	25.1	*	1.19	0.68		63	60
Proline	8.0	8.8	NS	3.2	3.7	NS	0.55	0.21		40	42
Asparagine	5.7	4.9	NS	1.6	1.9	NS	0.51	0.13		28	39
Glutamine	57.5	31	*	22.7	18.3	*	8.53	1.57		39	59
Lysine	12.1	10.9	NS	3.4	3.2	NS	1.16	0.25		28	29
Histidine	9.7	8	NS	7.4	3.6	*	0.53	0.48		76	45
Tyrosine	4.4	4.7	NS	1.6	1.5	NS	0.22	0.11		37	33
Tryptophan	0.7	0.6	NS	0.3	0.2	NS	0.02	0.01		41	36
β-Alanine	2.3	2.0	NS	1.9	1.2	NS	0.13	0.09		83	58
Total	247.0	196.0	*	121.0	99.2	*	18.61	4.89		49	51
*Sugars*											
Glucose	180	200	*	65	77	*	0.70	0.65		36	38
Mannose	32	30	NS	11.7	9.5	NS	0.04	0.01		37	32
Fructose	69	77	*	27.3	26	NS	0.03	0.02		39	34
Ribose	10	10	NS	3.2	3	NS	0.08	0.07		32	30
Maltose	13.5	11.3	*	4.8	4.2	*	0.06	0.06		35	37

^1^ Probability that there is a difference between means; NS, no significant difference between means (*p* > 0.05); * significant at the 5% level.

The concentrations of sugars (glucose, mannose, fructose, ribose, and maltose) in raw and cooked goat and lamb meat are listed in Table 2. The concentrations of sugars were similar in the two species, and have been reported in meat in general. The carbohydrate at the highest concentration in goat and lamb meat was glucose (180 and 200 mg/100 g, respectively), while ribose was the lowest (10 mg/100 g). Our results are similar to those obtained for sugars in lamb and goat, which have always shown glucose at the highest concentration, followed by fructose, with ribose at the lowest concentration. All sugar concentrations decreased with cooking.

### 2.3. Volatile Compounds

One hundred and thirty-three compounds were quantified in the headspace of the grilled meat from lamb and goat. These compounds, which comprised eighty-seven lipid-derived and forty-six Maillard-derived ones, consisted of 25 aldehydes, 25 ketones, 22 hydrocarbons, 16 alcohols, 11 pyrazines, 10 dimethyl sulphides, eight furans, seven nitrogen-containing compounds, four thiophenes, four acids and one thiazole. Of these compounds, 15 were reported for the first time in lamb or goat meat; however, these compounds have been previously reported in other meats. The concentrations of these compounds are grouped according to their functionality and are listed in Table 3.

**Table 3:** Volatile compounds in the headspace of the grilled *Longissimus dorsi* from Saanen goat and cross Suffolk lamb.

Compound	Mean concentration (ng/100 g) ^a^		*P* ^b^	Standard error	LRI ^c^	ID ^d^
	Goat	Lamb				
*LIPID-DERIVED COMPOUNDS*						
*Hydrocarbons*						
Pentane ^f,g^	10	2	*	1.34	500	A
3-Methylpentane ^e,g^	22	17	*	1.21	<600	B
Benzene ^f,g^	3	2	NS	0.17	657	A
Heptane ^f,g^	7	2	*	0.75	700	A
Toluene ^f,g^	17	14	NS	0.94	769	A
Octane ^f,g^	105	55	*	11.57	800	A
(*E*)-2-Octene ^f,h^	1	1	NS	0.19	814	B
(*Z*)-2-octene ^f,h^	2	3	NS	0.47	806	B
Ethylbenzene ^f,g^	2	1	NS	0.06	865	A
1,3-Dimethylbenzene ^f,g^	6	4	*	0.27	874	A
1,4-Dimethylbenzene ^f,g^	0.04	0.04	NS	0.00	876	A
Styrene ^f,g^	1	2	NS	0.09	897	A
1,2-Dimethylbenzene ^f,g^	2	2	NS	0.09	898	A
Nonane ^f,g^	4	2	NS	0.37	900	A
2,2,4,6,6-Pentamethylheptane ^e,g^	6	47	*	5.89	993	A
Decane ^f,g^	4	2	NS	0.55	1000	A
Limonene ^f,g^	2	1	NS	0.10	1037	A
Camphor ^e,h^	1	0.0	NS	0.10	1165	B
Dodecane ^f,g^	1	1	NS	0.05	1200	A
Tridecane ^f,g^	5	5	NS	0.74	1300	A
Tetradecane ^f,g^	1	1	NS	0.08	1400	A
Pentadecane ^f,g^	1	3	NS	0.30	1500	A
*Aldehydes*						
Pentanal ^f,g^	364	75	*	41.98	699	A
*(E)-*2-Methyl-2-butenal ^f,g^	1	1	NS	0.03	741	A
Hexanal ^f,g^	1943	252	*	244.29	802	A
Heptanal ^f,g^	319	127	*	33.89	903	A
*(E)-*2-Heptenal ^f,g^	4	0.2	NS	0.59	960	A
Benzaldehyde ^f,g^	30	34	NS	2.34	971	A
Octanal ^f,g^	229	94	*	24.24	1005	A
5-Ethyl-1-formylcyclopentene ^f,g^	2	0.1	NS	0.32	1041	C (1)
*(E)-*2-Octenal ^f,g^	7	0.2	NS	0.96	1061	A
(*Z)-*6-Nonenal ^e,h^	0	0.2	NS	0.04	1100	D (4)
Nonanal ^f,g^	378	210	*	40.11	1108	A
*(E)-*2-Nonenal ^f,g^	7	1	*	0.85	1164	A
2-Ethylbenzaldehyde ^f,g^	0.1	0.4	NS	0.08	1175	C [29]
Decanal ^f,g^	13	9	*	1.20	1208	A
5-Butyl-1-formylcyclopentene ^f,g^	0.06	0.01	*	0.01	1233	C [18]
*(E)*-2-Decenal ^f,g^	6	1	*	0.78	1266	A
Undecanal ^f,g^	3	1	*	0.44	1312	A
*(E,E)*-2,4-Decadienal ^f,g^	2	0.2	*	0.32	1327	A
*(E)-*2-Undecenal ^f,g^	6	1	*	0.67	1371	A
Dodecanal ^f,g^	1	0.5	*	0.11	1414	A
4-Pentylbenzaldehyde ^f,g^	0.2	0.1	NS	0.03	1480	B
*Ketones*						
2-Propanone ^f,g^	78	80	*	8.06	<600	B
2-Butanone ^f,g^	448	406	*	10.70	<600	B
2-Pentanone ^f,g^	19	14	*	0.79	683	A
3-Pentanone ^e,g^	7	6	NS	0.15	693	A
3-Hexanone ^f,g^	3	0.3	*	0.33	784	A
2-Hexanone ^f,g^	2	1	NS	0.32	788	A
Cyclopentanone ^f,g^	1	2	NS	0.11	794	A
4-Hydroxy-2-pentanone ^e,h^	1	0.0	NS	0.18	818	D [3]
2-Methylcyclopentanone ^f,g^	1	0.4	*	0.07	845	A
5-Methyl-2-hexanone ^e,h^	0.4	0.5	NS	0.02	856	A
2-Heptanone ^f,g^	33	11	*	3.46	890	A
3-Ethylcyclopentanone ^f,g^	2	1	NS	0.12	966	A
1-Octen-3-one ^f,g^	1	1	NS	0.18	978	B
2,3-Octanedione ^f,g^	28	6	*	3.73	983	A
3-Octanone ^f,g^	5	2	NS	0.41	986	B
2-Octanone ^f,g^	1	1	NS	0.06	990	A
2-Nonanone ^f,g^	3	3	NS	0.12	1091	A
2-Decanone ^f,g^	3	2	NS	0.14	1193	A
2-Undecanone ^f,g^	0.5	0.4	NS	0.02	1295	A
2-Tridecanone ^f,g^	0.1	0.1	NS	0.01	1497	A
*Alcohols*						
2-Butanol ^f,g^	41	31	*	1.71	<600	B
2-Methyl-1-propanol ^f,g^	2	8	NS	0.97	616	A
1-Butanol ^f,g^	84	5	*	12.27	654	A
1-Penten-3-ol ^f,g^	28	51	*	5.30	679	A
3-Methyl-3-buten-1-ol ^f,g^	2	1	NS	0.28	729	A
3-Methylbutan-1-ol ^f,g^	20	22	NS	0.90	736	A
1-Pentanol ^f,g^	142	51	*	13.69	765	A
1-Hexanol ^f,g^	37	20	*	2.58	867	A
1-Heptanol ^f,g^	3	2	NS	0.23	969	A
1-Octen-3-ol ^f,g^	124	77	*	7.10	980	A
3-Octanol ^f,g^	1	2	NS	0.26	997	A
2-Ethyl-1-hexanol ^f,g^	80	6	*	11.02	1028	A
2-Octen-1-ol (*E* and /or *Z*) ^f,g^	9	3	NS	0.96	1067	A
1-Octanol ^f,g^	49	26	*	4.87	1069	A
1-Nonanol ^f,g^	3	1	NS	0.37	1163	A
1-Dodecanol ^f,g^	2	4	NS	0.44	1462	B
*Acids*						
Hexanoic acid ^f,g^	4	1	*	0.37	964	A
Octanoic acid ^f,g^	0.3	0.2	NS	0.02	1157	A
Nonanoic acid ^f,g^	0.3	0.2	NS	0.02	1254	A
*Furans*						
2-Ethylfuran ^f,g^	2	1	NS	0.20	698	A
2-Butylfuran ^f,g^	0.4	0.1	NS	0.05	893	A
2-Pentylfuran ^f,g^	14	3	*	1.70	992	A
2-Hexylfuran ^f,g^	0.1	0.1	NS	0.01	1092	A
2-Octylfuran ^f,g^	0.5	0.1	NS	0.06	1297	A
*MAILLARD-DERIVED COMPOUNDS*						
*Nonheterocyclic*						
2-Methylpropanal ^f,g^	92	69	*	4.71	<600	B
3-Methylbutanal ^f,g^	141	107	*	4.93	649	A
2-Methylbutanal ^f,g^	129	92	*	5.77	660	A
Benzeneacetaldehyde ^f,g^	2	1	NS	0.16	1052	A
2.3-Butanedione ^f,g^	34	20	*	2.08	<600	B
2,3-Pentanedione ^f,g^	1	1	NS	0.09	695	A
2,4-Pentanedione ^e,h^	0.2	0.2	NS	0.03	778	A
3-Hydroxy-2-butanone ^f,g^	21	3	*	2.96	708	A
3-Metylbutanoic acid ^f,g^	5	5	NS	0.55	827	B
*Sugar-derived*						
2-Furfural ^f,g^	0.4	0.4	NS	0.02	834	A
2-Furanmethanol ^f,g^	0.2	0.2	NS	0.02	853	A
2-Acetylfuran ^f,g^	1	0.8	NS	0.06	913	A
*Nitrogen heterocyclic*						
*N*-Methylpyrrole ^f,g^	3	2	NS	0.23	738	A
Pyrrole ^f,g^	12	10	NS	0.97	747	A
Pyridine ^f,g^	1	1	NS	0.16	748	A
1-Ethyl-1*H*-pyrrole ^e,h^	1	1	NS	0.08	814	C [18]
3-Methyl-1-*H*-pyrrole ^e,h^	0.3	0.3	NS	0.03	836	C [18]
2-Methyl-1-*H*-pyrrole ^f,g^	0.4	0.3	NS	0.03	844	C [18]
Indole ^f,g^	2	0.4	*	0.58	1309	D [2]
*Pyrazines*						
2-Methylpyrazine ^f,g^	4	2	*	0.34	828	A
2,5(6)-Dimethylpyrazine ^f,g^	14	8	*	0.97	916	A
Ethylpyrazine ^f,g^	1	0.4	NS	0.06	922	A
2-Ethyl-6-methylpyrazine ^f,g^	5	3	*	0.36	1001	A
Trimethylpyrazine ^f,g^	12	6	*	1.00	1005	A
2-Ethyl-5-methylpyrazine ^f,g^	8	5	NS	0.52	1007	A
3,6-Dimethyl-2-ethylpyrazine ^f,g^	12	8	*	0.67	1080	A
3,5-Dimethyl-2-ethylpyrazine ^f,g^	2	1	NS	0.19	1086	A
2,3-Dimethyl-5-ethylpyrazine ^f,g^	1	1	NS	0.14	1090	A
2,5-Diethyl-5-methylpyrazine ^f,g^	0.06	0.04	NS	0.00	1156	B
2,3,5-Trimethyl-6-ethylpyrazine ^e,h^	1	0.5	NS	0.09	1160	C [2]
*Dimethyl sulfide*						
Hydrogen sulphide ^f,g^	19	19	NS	0.61	<600	B
Sulfur dioxide ^f,g^	1	1	NS	0.08	<600	B
Methanethiol ^f,g^	4	4	NS	0.11	<600	B
Carbon disulfide ^f,g^	3	3	NS	0.09	<600	B
Ethylmethylsulfide ^e,h^	0.3	0.4	NS	0.02	604	B
Dimethyl disulfide ^f,g^	4	3	NS	0.25	745	A
Ethylmethyldisulfide ^e,g^	0.02	0.02	NS	0.00	840	B
Methional ^f,g^	0.06	0.06	NS	0.00	910	A
Dimetyl trisulfide ^f,g^	1	1	NS	0.06	981	A
Dimethyl tetrasulfide ^f,g^	0.07	0.07	NS	0.02	1241	C [29]
*Thiophenes*						
Thiophene ^f,g^	3	3	NS	0.16	666	A
2-Methylthiophene ^f,g^	1	1	NS	0.08	773	A
3-Methylthiophene ^f,g^	0.4	0.4	NS	0.02	783	A
2-Ethylthiophene ^f,g^	0.1	0.1	NS	0.00	866	A
*Thiazoles*						
5-Mehtylthiazole ^f,g^	0.09	0.04	*	0.01	820	A
*Miscellaneous*						
Acetophenone ^f,g^	1	2	NS	0.09	1075	A

^a^ The means are from four replicate samples; ^b^ Probability that there is a difference between means; NS, no significant difference between means (*p* > 0.05); * significant at the 5% level; ^c^ Linear retention index on a CP-Sil 8 CB low bleed/MS column; ^d^ A, mass spectrum and LRI agree with those of authentic compounds; B, mass spectrum identified using NIST/EPA/NIH Mass Spectral Database and LRI agrees with literature value [31], C, Mass spectrum agrees with spectrum in NIST/EPA/NIH Mass Spectral Database or with other literature spectrum [2,3,18,19,29]; D, Tentative identification where mass spectrum agrees with reference spectrum in the NIST/EPA/NIH mass spectral database [2,3]; ^e^ Reported for the first time in cooked goat; ^f^ Previously reported in grilled goat [2,3,19]; ^g^ Previously reported in cooked lamb; ^h^ Reported for the first time in cooked lamb*.*

The number of compounds was the same for both species; on the other hand, differences were observed between the total amounts (ng/100 g) of volatiles in goat and lamb meat. The level for lamb meat was 2.4-fold greater than for goat meat. Of the 133 identified compounds, 99 were affected by species, 75% of which were lipid-derived compounds. Additionally, 85 were found in higher concentrations in lamb, against the 14 compounds found in goat headspace. The latter included five alcohols, four hydrocarbons, three ketones, and two aldehydes.

Aldehydes, ketones and alcohols were quantitatively the dominant classes of volatiles in the cooked goat and lamb; however, the concentration of aldehydes was 4.1-times greater in lamb than in goat. The most abundant volatiles were hexanal, 2-butanone, nonanal, heptanal and octanal.

The compounds formed by the Maillard reaction were not different by species, except for the pyrazines and Strecker aldehydes, benzeneacetaldehyde, 2,3-butanedione, 3-hydroxy-2-butanone, and 5-methylthiazole, which were at the highest concentrations in lamb meat. Sugar-derived compounds were not different in the volatile profiles of goat and lamb meat. However, the concentrations of aldehydes, ketones, alcohols, acids, and furan, all volatiles derived from the oxidation of fatty acids, were very different in lamb and goat. In general, 86% of aldehydes, 69% of alcohols, 65% of ketones, and 54% of hydrocarbons were found in the highest concentrations in lamb. These data reflect the differences in fatty acid profile of these meats, in which the highest concentrations of fatty acids were detected in lamb meat, as discussed earlier. Elmore *et al.* [19] found that changes in the fatty acid composition of lamb and beef resulted in changes in the volatile composition [20]. They reported that cooked beef and lamb samples that contained increased levels of PUFAs had higher concentrations of lipid oxidation products, particularly saturated and unsaturated aliphatic aldehydes.

The high level of linoleic acid in lamb meat accounted for the high levels of volatiles reported in the literature [18]. These volatiles included the oxidation products of C18:2*n*6, e.g., hexanal, pentanal, octanal, and heptanal, 1-pentanol, 2-octen-1-ol, 1-octen-3-ol, 1-hexanol, and 2-pentylfuran, all detected at concentrations greater than 10 ng/100 g. Similarly, 1-penten-3-ol, 2-ethylfuran, benzaldehyde, 2-ethylbenzaldehyde, and *Z*-6-nonenal, which were reported to be the oxidation products of α-linolenic acid [19], were detected in high concentrations in goat meat, which also contained a high concentration of this fatty acid.

Alkylpyrazines were the major class of Maillard-derived volatiles and were 1.8-fold higher in goat meat than in lamb. The highest concentrations of pyrazine in goat meat most likely resulted from the fact that this species had the highest concentration of free glycine. Low *et al.* [32] have demonstrated that glycine plays a major role promoting the formation of 2,3-dimethylpyrazine, 2-ethyl-3,5-dimethylpyrazine, trimethylpyrazine, and 2,5-diethyl-3-methylpyrazine. 5-methylthiazole, which is formed from the lipid-Maillard interaction, was also found at the highest concentrations in goat meat. The highest concentrations of pyrazines and thiazole in goat meat may intensify the roasted and overall flavour of goat meat. There was clear evidence that concentrations of the sulphur-containing Maillard-derived volatiles, dimethyl sulphides and thiophenes, were not different between goat and lamb; consequently, their contribution to the basic meaty flavour appears to be similar.

The compounds that showed the greatest differences between goat and lamb meat, e.g., the C_6_-C_9_ *n*-alkanals and C_4_-C_5_ *n*-alcohols, 1-octen-3-ol, and 2-butanone, are likely to have the biggest effect on flavour differences between goat and lamb. Mottram [33] explained that species differences in flavour are largely explained by differences in lipid-derived volatile components. These compounds are important contributors to the mutton/goat odour of sheep and goat.

## 3. Experimental

### 3.1. Species

The goat breed Saanen, a widespread breed used for milk production, and the sheep breed Suffolk lamb were used in this study. The animals were vaccinated and dewormed and then placed into 0.80 × 1.20 m individual stalls with free access to food (hay and straw) and water. The acclimation period was 14 days, with a maximum of 56 days before slaughter; the male goats were approximately 5–6 months old, and the male lambs were 9 months old. The animals were slaughtered, according to European Union regulations, after fasting for 18 h; water was available during the fasting period. The carcasses were dressed, and stored at 4 °C for 7 days. Subsequently, steaks were prepared, vacuum packed and stored at −18 °C, for a period no longer than 30 days before analysis. A portion of the left and right *Longissimus dorsi* of four animals from each species was studied.

### 3.2. Grilling

Sixty *Longissimus dorsi* chops, thirty from each species, were grilled, to analyse the volatile profile and the concentration of some water-soluble precursors of the cooked meat. When required for analysis, the chops, which had been stored at −18 °C, were defrosted overnight at 4 °C, cleaned of any visible fat, and cooked to a coretemperature of 80 °C, measured using a thermocouple type K, mode 3200K (Digitron Instrumentation Ltd., Devon, UK). The chops were grilled using a two-plate grill (CUISINART Griddle & Grill – GR4U, Wigan, UK).

Cooking to a fixed temperature compensated to an extent for any variations in the thickness and weight between and within the samples, although thicker chops took longer to cook and hence received more surface heat than thinner ones. Samples ranged in weight from 113 to 195 g, in thickness from 17 to 30 mm, and in grilling time from 6 to 12 min. Samples were weighed directly before and immediately after cooking to measure moisture losses during the grilling process, so that changes in the concentrations of non-volatile components during grilling could be measured independently of changes due to moisture content. In addition, to compensate for any bias in volatiles and precursors analysis, each goat or lamb chop was grilled and analysed together, with one goat sample followed by a lamb sample, consecutively.

Directly after cooking, the grilled chop was left to cool to 30 °C to 35 °C, and then was minced in an electric bowl chopper. The aromatic volatiles were extracted from grilled meat using headspace concentration on Tenax and were analysed using gas chromatography-mass spectrometry. For each grilled *Longissimus dorsi* chop, four replicate analyses were performed.

### 3.3. Analysis of Volatile Compounds

Reagents and chemicals were purchased from normal laboratory suppliers (Merck, Frankfurt, Germany) and were of analytical grade. Volatiles were extracted by dynamic headspace entrainment on Tenax TA. The minced grilled meat (20 ± 0.1 g) was placed in a screw-top conical flask (250 mL). A Dreschel head was attached to the flask using an SVL fitting (Bibby, Stone, UK). The flask was held in a water bath at 60 °C for 1 h, while oxygen-free nitrogen at 40 mL/min swept the volatiles onto a pre-conditioned glass trap (4 mm i.d., ¼" o.d. × 3.5 mm long), packed with Tenax TA (Supelco, Poole, UK). A standard (130.6 ng 1,2-dichlorobenzene in 1 µL methanol was added to the trap at the end of the collection and excess solvent and any water retained on the trap was removed by purging the trap with nitrogen at 40 mL/min for 10 min [18,19].

Volatile analyses were performed on a Perkin-Elmer Clarus 500 GC-MS system (Perkin-Elmer, Beaconsfield, UK), equipped with an automated thermal desorber (Turbomatrix ATD), and TurboMass software (Version 4.5, Perkin-Elmer). The Tenax tubes were desorbed at 300 °C (heating rate 40 °C/s) and cryofocused onto a packed cold trap at −30 °C. GC separation was carried out on a DB-5 nonpolar column (60 m × 0.32 mm I.D., 1 μm film thickness, J & W Scientific). The temperature program employed was 2 min at 40 °C with a ramp of 4 °C/min to 280 °C, and held for 10 min. Helium at 16 psi was used as the carrier gas, resulting in a flow of 1.0 mL·min^−1^ at 40 °C. A series of *n*-alkanes (C_6_- C_25_) in diethyl ether was analysed under the same conditions to obtain linear retention index (LRI) values for the volatile compounds [2].

The mass spectrometer operated in electron impact mode with a source temperature of 230 °C, an ionising voltage of 70 eV, and a scan range from *m/z* 29 to *m/z* 300 at 2.76 scans/s. Compounds were identified by first comparing their mass spectra with spectra from authentic compounds previously analysed [33] spectra from the NIST/EPA/NIH Mass Spectral Database (Version 2.0a, 2002), or spectra published elsewhere. Wherever possible, identities were confirmed by comparison of linear retention index (LRI) values, with either those of authentic standards or published values. The approximate quantities of the volatiles were estimated by comparison of their peak areas with that of the 1,2-dichlorobenzene internal standard obtained from the total ion chromatograms, using a response factor of 1 [18,19].

### 3.4. Fatty Acid Analysis

The fatty acids of goat and lamb meat were extracted, methylated, and analysed by gas chromatography as described by [34]. The lipids were extracted from the meat (2 g) using chloroform:methanol (2:1), and nonsaponifiables were removed by the addition of 0.73% NaCl. The fatty acids were methylated with a solution of methanolic sodium methoxide [35]. The FAME were separated using a gas chromatograph (GC, 3400 Varian Inc., Palo Alto, CA, USA) equipped with a flame ionisation detector, automatic injector, split injection port, and a 100 m fused silica capillary column (i.d. 0.25 mm, CP-SIL 88, Varian Inc.) with hydrogen as the carrier gas. A split injection of 50:1 was used, with the injector at 255 °C. After 4 min at 70 °C, the column temperature was raised at 8 °C/min to 110 °C, raised to 170 °C at a rate of 5 °C/min, held at 170 °C for 10 min, and increased at 4 °C/min to a final temperature of 240 °C that was maintained for 14.5 min. The carrier gas was hydrogen at a flow rate of 2.1 mL/min. The GC peaks were identified using authentic FAME standards (GLC 463; UC-59-M, Nu-Chek-Prep Inc., Elysian, MN; P9125, O4754, O9881, E4762, V1381, Sigma-Aldrich Company Ltd, Dorset, UK). Fatty acids were quantified using heneicosanoic acid methyl ester as an internal standard added prior to methylation. Duplicate analyses were performed on all goat and lamb samples, and the meat FA results were expressed as mg/100 g total FAME.

### 3.5. Preparation of Sample Extracts for Analysis of Water-Soluble Compounds (Free Amino Acids and Sugars)

Portions of 2.5 g ± 0.01 g of the minced raw or grilled goat or lamb meat were weighed into polypropylene copolymer NalgeneR Oak Ridge centrifuge tubes (Nalge Nunc International, Rochester, NY, USA). Cold water (10 mL) was added to the tubes. The samples were emulsifying by vortexing for 3 min prior to centrifugation at 8,000 *g* for 30 min at 4 °C in a RC-5C Plus SorvallR centrifuge. The supernatant was filtered under vacuum through Whatman N° 1 qualitative filter paper. Finally, 400 μL of the filtrate was transferred to an Ultrafree-MC ultra filtration tube with 3000 MWCO regenerated cellulose membrane (Millipore Corp., Bedford, MA, USA), and centrifuged at 7,200 g for 30 min. The filtrate was stored at −18 °C until the analysis of free amino acids [36].

For the analysis of sugars, portions of 0.5 g of raw or cooked minced goat or lamb meat were weighed and transferred to a 14 mL glass bottle, to which was added 10 mL of cold HPLC water. The contents of the bottle were emulsifying by vortexing for 1 min prior to stirring for 15 min. The samples then were allowed to stand for 15 min. The supernatants (1.5 mL) were transferred to Sep-Pak Plus C18 cartridges (Waters Corp., Milford, MA, USA) and centrifuged at 7200 g for 15 min. Finally, 450 μL of supernatant was mixed with 50 μL trehalose solution (20 μL/100 mL) before analysis by anion exchange chromatography. Raw or cooked goat or lamb left or right *Longissimus dorsi* from each animal were extracted in triplicate for analysis of water-soluble components.

### 3.6. Determination of Free Amino Acids by GC-MS

The free amino acids were analysed by CG/MS using the EZ-Faast amino acid derivatisation technique (Phenomenex, Macclesfield, UK), followed by analysis on the Agilent 5975 GC-MS system (Agilent, Santa Clara, CA, USA) as described by Elmore *et al.* [36].

### 3.7. Determination of Monosaccharides and Disaccharides by Anion Exchange Chromatography HPIC-PAD

The determination of monosaccharides and disaccharides was carried out following the method described by Elmore *et al.* [37], on an 8220i Dionex high-performance anion exchange chromatography system (HPIC-PAD) with pulsed amperometric detection (Dionex Corp., Sunnyvale, CA, USA). The ion chromatography system consisted of an AS50 autosampler, an LC25 column oven, GS50 pumps, and an ED50 pulsed amperometric detector, running in internal amperometric mode [37].

### 3.8. Statistical Analysis

The data were compiled into spreadsheets and analyzed by Student’s t test at 5% significance. Analyses were performed with aid of the SAS statistical software version 9.1.3 [38].

## 4. Conclusions

Differences in the content and properties of intramuscular fatty acid profile between species are an important factor explaining the differences in volatile profiles. Compared to goat, lamb meat had higher levels of SFA, MUFA and PUFA and similar levels of sugars and free amino acids, except for lysine and glycine, which were highest in goat. Major differences were detected in lipid-derived volatiles, as only pyrazine, thiazole, and some Strecker aldehydes were different in concentration between these species. Volatile compounds derived from the oxidation of linoleic acid were at higher levels in meat from lamb due to the higher concentration of this acid, while compounds formed from α-linolenic acid were more intense in goat. It can be concluded that lamb meat most likely has a stronger flavour profile compared to goat meat because it had the highest concentration of lipid-derived volatile compounds, pyrazines and thiazole.
